# Strategic design of a Mussel-inspired in situ reduced Ag/Au-Nanoparticle Coated Magnesium Alloy for enhanced viability, antibacterial property and decelerated corrosion rates for degradable implant Applications

**DOI:** 10.1038/s41598-018-36545-3

**Published:** 2019-01-15

**Authors:** Abdelrahman I. Rezk, Arathyram Ramachandra Kurup Sasikala, Amin Ghavami Nejad, Hamouda M. Mousa, Young Min Oh, Chan Hee Park, Cheol Sang Kim

**Affiliations:** 1 Department of Bionanosystem Engineering, Graduate School, Chonbuk National University, Jeonju, Jeonbuk 561-756 Republic of Korea; 2 Department of Engineering Materials and Mechanical Design, Faculty of Engineering, South Valley University, Qena, 83523 Egypt; 3 Division of Mechanical Design Engineering, Chonbuk National University, Jeonju, Jeonbuk 561-756 Republic of Korea; 4 Department of Neurosurgery, Chonbuk National University Medical School & Hospital, Chonbuk National University, Jeonju, Jeonbuk 561-756 Republic of Korea

## Abstract

Magnesium (Mg) and its alloys have attracted much attention as a promising candidate for degradable implant applications however the rapid corrosion of magnesium inside the human body greatly limits its use as an implant material. Therefore, coating the alloy surface with a multifunctional film is a promising way to overcome the drawbacks. Here we propose for the first time a multifunction layer coating to enhance the cell viability, antibacterial property and decelerated corrosion rates to act as a novel material to be used for degradable implant Applications. For that, the magnesium alloy (AZ31) was first treated with hydrofluoric acid (HF) and then dopamine tris Hydrochloric acid (tris-HCL) solution. The reducing catechol groups in the polydopamine (PD) layer subsequently immobilize silver/gold ions *in situ* to form uniformly dispersed Ag/Au nanoparticles on the coating layer. The successful formation of Ag/Au nanoparticles on the HF-PD AZ31 alloy was confirmed using XPS and XRD, and the morphology of all the coated samples were investigated using SEM images. The alloy with HF-PDA exhibit enhanced cell attachment and proliferation. Moreover, the nanoparticle immobilized HF-PD alloy exhibited dramatic corrosion resistance enhancement with superior antibacterial properties and accountable biocompatibility. Thus the result suggest that HF-PD Ag/Au alloy has great potential in the application of degradable implant and the surface modification method is of great significance to determine its properties.

## Introduction

In the last few years, magnesium (Mg) and its alloys have attracted much attention as a promising candidate for degradable implant applications such as bone-fixation plates, screws, wires, pins, and stents^1^, all of which are commonly used during surgical operations such as orthopedic and angioplasty interventions to assist in the healing period and maintain the structural support^2^. In comparison with other metals, for instance, Titanium and iron are mainly used as implant materials. Titanium is more inert and stable in the human body but is quite expensive and need further surgery after healing process while iron are more economic however, it’s comparatively more reactive when compared to titanium^3,4^. Co-Cr alloys were also tried but result in adverse host response and acute toxicity. Mg is highly biocompatible and can naturally dissolve *in vivo*^5,6^. Furthermore, Mg has comparable physical characteristics regarding natural bone such as Young’s modulus and tensile strength that make it a suitable candidate for implant applications^7,8^. Although Mg and its alloys can be effectively used in orthopedic temporary-implant applications, they are also susceptible to an accelerated degradation in aqueous environments^9,10^. To date, extensive research efforts have been directed toward controlling the degradation rate of Mg and its alloys^11–13^. In most cases, the interfacial characteristics and the interfacial interactions between implants and their surrounding tissue can determine the success of the implantation^14^. The corrosion rate, osteointegration, and bacterial infection have been identified from among the interfacial processes as the most pertinent in terms of the improvement of new materials and devices for temporary-implant applications^15,16^. Over the last two decades, several studies have dedicated great effort toward the enhancement of the corrosion resistance and osteointegration of implants by using an Mg coating with different polymers or nanoparticles (NPs)^17–19^. Learning from nature is endless source of inspiration. In the present section, universal coatings that have been inspired or directly collected from natural biological systems of blood proteins, mussel foot proteins, and plant phenols will be described and discussed. These bioinspired surface coatings bind to substrate surfaces by multiple combined interactions, besides simple chemisorption or physisorption, to enhance the stability of the coatings under different conditions.

Among the different polymers, biomimetic polymers such as mussel-inspired polydopamine (PD) are considered as one of the most attractive types of material for the treatment of metallic alloys to improve the corrosion behavior^20^. Dopamine is one of the most essential human neurotransmitters, and its ability to form a biocompatible coating is advantageous^21,22^, along with the advantage of its easy polymerization process in alkaline environments^22^. Because of this alkaline-polymerization process, PD is an attractive material for a direct-coating formation during the polymerization for which an Mg alloy is immersed in an aqueous solution, as the alkaline solution can minimize the Mg corrosion during the coating formation. Many studies have focused on PD layer^23^, reporting that the corrosion-current density of PD-coated Mg is lower compared with the bare alloy. Also, several studies have shown that the polydopamine modification of bioceramics significantly promoted the attachment and proliferation of cells due to the improved surface roughness, hydrophilicity, and bioactive functional groups (e.g. OH− and NH_2_−) of PDA^24,25^. Moreover, the improvement of apatite mineralization of PDA bioceramics may be the important reason to enhance the adsorption of serum proteins and further improve attachment, proliferation, differentiation and bone-related gene expression of cells^25^. From these findings, it was proved that PD comprises effective adhesive, cytocompatibility, and anticorrosive activities among the organic and inorganic coatings. Another unique property of a formed PD layer is a strong reductive property. The catechol-PD group is capable of reducing metal ions such as those of silver (Ag) and gold (Au) into NPs^22^. Therefore, it should be possible to take this advantage to form NPs on the surface of a PD-coated substrate by simply immersing the materials in a dilute metal-ion solution.

Ag or Au NPs displayed many characteristics make them suitable for different applications such as photocatalysis and biosensors^26–29^. Also there are reports suggesting the diversified application of gold nanoparticles as memory device^30,31^ and composites of gold with other nanoparticles for photocatalytic and other applications^32–34^. Apart from these the gold and silver nanoparticle exhibit excellent antibacterial property^35^. A high infection rate at the bone-implant site has been considered as a major risk accompaniment of medical-implant processes. Therefore, coating an implant with Ag or Au NPs had been shown great benefits due to their antimicrobial, antioxidative, and anticorrosive properties. Zhang *et al*. studied the Au-NP efficiency against gram-negative and gram-positive bacteria, as Au NPs inhibit the growth and colonization of different kinds of microbes^36,37^, while reports state that Ag-NP coatings have displayed excellent antibacterial properties that are capable of addressing the bacterial multiplication on implants and other nosocomial infections during surgical procedures^38,39^. The efficiency of the Ag and Au coatings in controlling the colonization of bacteria around an implant is dependent on the balance between the activity of the cytotoxic metal cation and the concentration of the ions released from the coating^36^. The high concentration of the cations that are released from the coating can be toxic to cells.

This study is devoted to investigate the effects of Ag and Au NPs enriched PD-hydrofluoric acid (HF)-coated AZ31 alloy, to retard the corrosion of the alloy with improved antibacterial effect with an accountable cytocompatibility under the physiological condition. The successful coating of the HF-treated AZ31 alloy with PD and Ag or Au NPs was confirmed using X-ray photoelectron spectroscopy (XPS) and X-ray diffraction (XRD), and the morphology was investigated using scanning electron microscope (SEM) images. The effectiveness of the polyelectrolyte-multilayer films on the HF-treated Mg alloy was evaluated in terms of the potentiodynamic-polarization test, the *in vitro* cytocompatibility, and the antibacterial test. The data of the present study indicate that the introduced materials display a sound biocompatibility and enhanced the corrosion resistance. Furthermore, the results revealed that this kind of coating not only improved the corrosion resistance, but it also released the Ag and Au NPs from the alloy surface, thereby providing an excellent antibacterial effect against both gram-positive and gram-negative bacteria species with a minimum toxicity against mammalian cells. The balance between the antibacterial effect and the cell response in consideration of the degradation process makes it an alternative in the application of the surface modification of the Mg alloy in degradable implant applications, and it is anticipated that the proposed surface-modification method can greatly contribute towards the progress of the development of Mg-alloy for medical devices.

## Results and Discussion

### Surface characterization and phase composition

**Figure 1:**
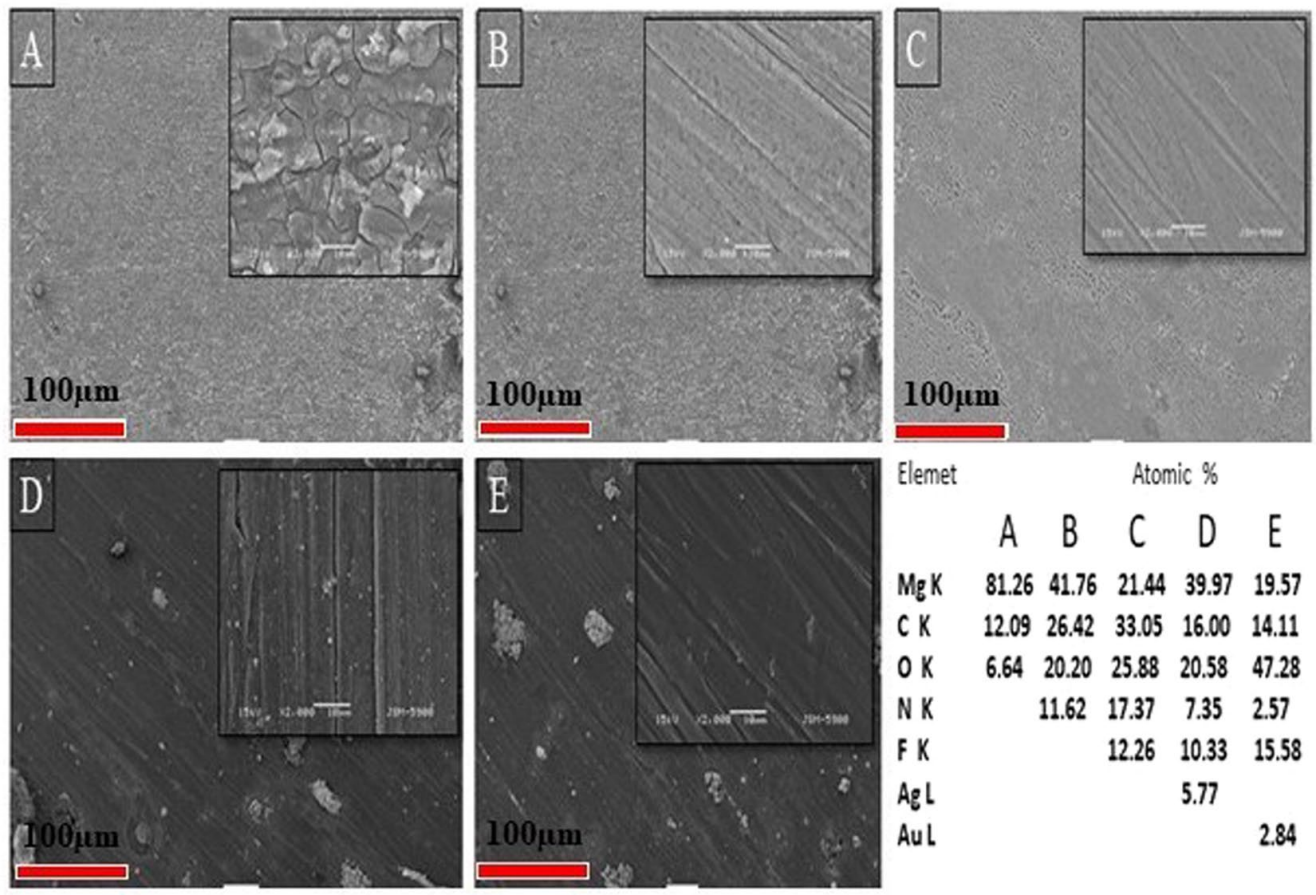
SEM images showing morphology of (**A**) bare alloy, (**B**) is PD-coated, (**C**) HF-pre-treated and PD-coated (HF-PD), (**D**) Ag HF-PD sample, and (**E**) Au HF-PD sample.

The characteristic morphology of the coated samples was analyzed using SEM (Fig. 1(A–E)). The SEM image shows that the morphology of bare alloy possesses a passive layer on the surface with cracks and pores; these defects could be unfavorable for long-term corrosion protection, as the corrosive media could penetrate through these defects, leading to an acceleration of the contact between the surface and the corrosive media. However, the PD modified surface, exhibits a homogenous layer of PD covering on the alloy surface to confirm the PD coating. In the HF-PD treated samples, a relatively smoother and crack-free surface has been obtained due to the simultaneous formations of PD and magnesium hydroxide Mg (OH)_2_ during the coating process. Figure 1(D,E) displayed relatively rougher surfaces with the uniform distribution of small particles on the samples, which indicate the successful in- situ reduction of the metal ions to the metallic NPs by polydopamine in the absence of any reducing agents. The uniform formation of PD on the alloy surface resulted in the synthesis of monodispersed NPs with a minimum degree of aggregation, which is expected to produce a strong antimicrobial effect. The successful coating of PD and the deposition of Ag and Au particles onto the surface were further confirmed using the EDX technique (inset table in Fig. 1) as well as the EDS elemental mapping (Fig. S1 of Supporting Information). Further confirmation of the formation of nanoparticles were carried out using FESEM (Fig. S2 of Supporting Information) and average size has been found to be 168.36 ± 48.5, and 151.03 ± 31.4 for Ag and Au particles respectively.

**Figure 2:**
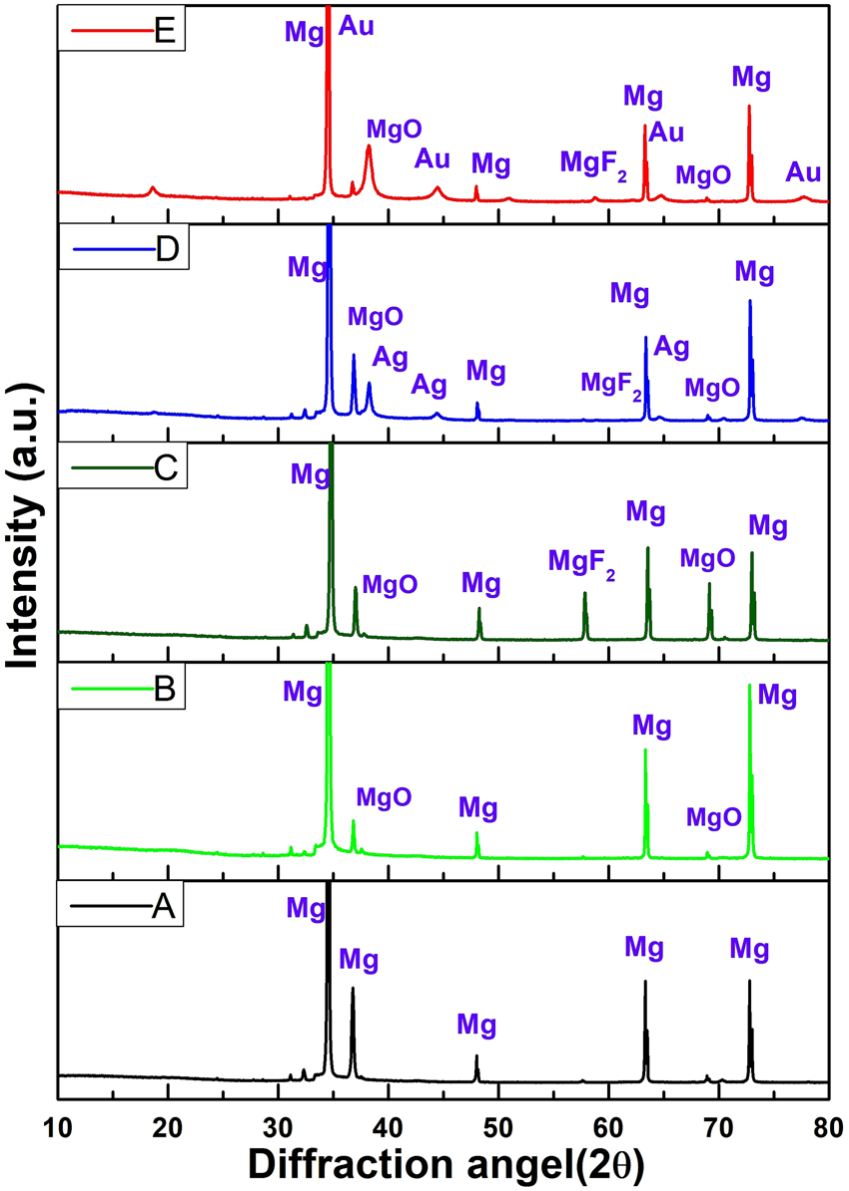
XRD patterns of (**A**) Bare alloy, (**B**) PD coated sample, (**C**) HF- PD coated Sample, (**D**) Ag HF-PD sample, and (**E**) Au HF-PD sample.

Phase analyses of the bare AZ31 alloy and the coated samples were analyzed using XRD (Fig. 2). The bare alloy display only Mg peaks whereas the PD coated and HF PD coated samples show the MgO and MgF_2_ peaks^40,41^_._ This is due to the formation of passive oxide layer, and MgF_2_ on the surface of bare alloy due to HF acid treatment as described below^41^:

1 $${{\rm{Mg}}}^{+2}+{{\rm{O}}}^{-{\rm{2}}}\to {\rm{MgO}}$$

2 $${{\rm{Mg}}}^{+2}+{{\rm{2F}}}^{-}\to {{\rm{MgF}}}_{{\rm{2}}}$$

The XRD patterns of the Ag- and Au-containing samples were also checked to confirm the formation of the NPs on the surface of the alloy. The Ag peaks were detected at 38.1°, 44.45°, and 63.5° while for Au-containing samples peaks detected at 38.1°, 44.38°, 63.5°, and 77.8°. The detected peaks correspond to the (111), (200), (220), and (311) diffractions of the metallic NPs (JCPDS No. 04–0783).

**Figure 3:**
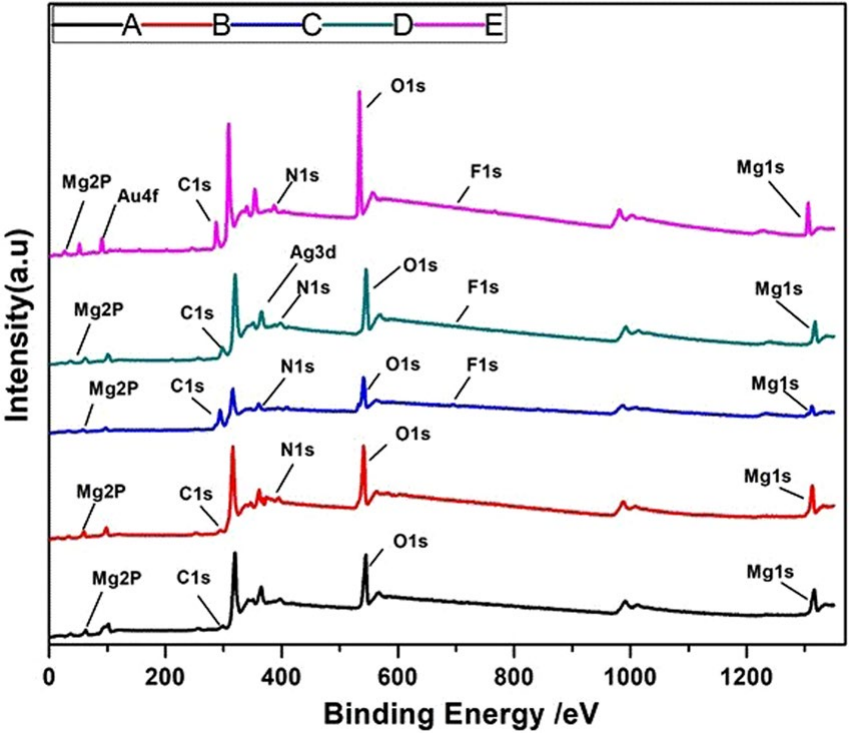
XPS full spectra and narrow-scan spectra of Ag and Au elements. (**A**) Bare alloy, (**B**) PD coated sample, (**C**) HF- PD coated sample, (**D**) Ag HF-PD sample, and (**E**) Au HF-PD sample.

The chemical compositions of the different coatings on the Mg-alloy substrate surface were characterized via the XPS analysis (Fig. 3). The full XPS spectra for the bare sample show the dominant presence of the Mg1s at 1303.54 eV, the Mg2p at 51.42 eV, and the O1s peaks at 530.18 eV, all of which are related to the Mg oxides. After the coating of the Mg alloy with PD, C1s and N1s peaks appeared for the samples and also the HF- PD treated sample exhibit an extra F1s peak at 692.16 eV (Fig. S3 of the Supporting Information). The XPS scan for the Ag- and Au-containing samples also detected the characteristic phases of both Ag and Au elements in their surface compositions. As shown in Fig. 3, the characteristic Ag3d peak at 367 eV corresponds to the bonding energy of the elemental Ag, and the Au4f was also detected at 95 eV, indicating the successful formation of the Ag and Au particles onto the surface of the coated alloy.

### Potentiodynamic test

**Figure 4:**
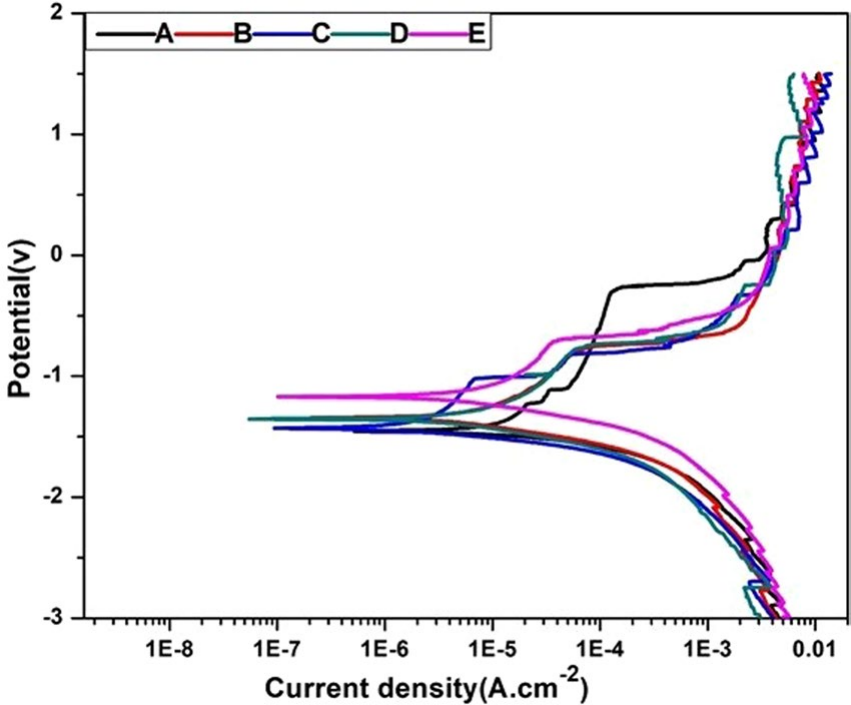
Potentiodynamic-polarization curves of a bare alloy and different coated samples. (**A**) Bare alloy, (**B**) PD coated sample, (**C**) HF- PD coated sample, (**D**) Ag HF-PD sample, and (**E**) Au HF-PD sample.

The degradation behavior of the Mg alloy was assessed using different methods. The potentiodynamic test is an essential test to determine the corrosion resistance of Mg and its alloys. The difference between the metallic potentials of the bare samples and the coated ones is an indication of the corrosion-resistance improvement. Herein, Fig. 4 shows the potentiodynamic-polarization measurements of the bare AZ31 alloy, PD coating, HF PD, and Composite-Ag/Au NPs on the HF-pre-treated PD-coated samples. Magnified images of the potentiodynamic-polarization measurements is given in Fig. S5 of the Supporting Information. The SBF solution was used as an electrolyte to mimic the physiological environment condition, and the solution was prepared according to a previous work^42^. The obtained results such as the corrosion potential, corrosion-current density, and corrosion rates were calculated and are also listed in Table 1 comparing to the previous coating materials and techniques of the magnesium interface. The different values were calculated using the commercial software ZIVE SM Smart Manager Ver. 5.503. In the electrochemical polarization test, the Mg corrosion potential (E_corr_) explains the relation between the cathodic and anodic reactions during the test, as the anodic one represents the Mg dissociation and the cathodic one displays the hydrogen (H_2_) evolution^43,44^. The test results are summarized in Table 1, and the results show that the corrosion-current densities for all of the coated samples are much lower than those of the bare alloy. The values were decreased by means of a dopamine formation on the alloy interface, which also indicates a successful formation of the PD layer. The surface pre-treatment with HF before the addition of the dopamine more retarded the degradation due to the formation of MgF_2_, which is more stable than Mg(OH)_2_ in the corrosive media. Moreover, the loading of the Ag and Au NPs on the surface improved the corrosion resistance, as the corresponding polarization-current densities are lower than those of the bare alloy and the HF-PD coated alloy. Thus the potentiodynamic polarization revealed that the corrosion resistance of the coated samples was significantly improved.

**Table 1:** Electrochemical Parameters of the Potentidynamic Polarization Curve of Different Samples in an SBF Solution compared to previous studies for AZ31 alloy.

Coating Material	Coating Technique	Corrosion properties			Immersion solution	Antibacterial Effect	Ref.
		E_Corr_ (V)	I_corr_ (µA/cm^2^)	CR (mmPY)			
Bare AZ31	None	−1.48	25.137	82.24	SBF	N/A	This study
Bare-PDA	Dip coating	−1.37	5.6	18.34	SBF	N/A	This study
HF-PDA	Dip coating	−1.45	3.189	10.43	SBF	N/A	This study
HF-PDA-Ag	Dip coating	−1.33	2.33	7.61	SBF	Ag	This study
HF-PDA-Au	Dip coating	−1.17	2.47	8.07	SBF	Au	This study
HA	Aerosol deposition	−1.56	4.76	N/A	SBF	N/A	^54^
Zn-Ca coating	Dip coating	−1.49	11.5	N/A	3.5 wt% NaCl	N/A	^55^
BTSE-coated	Dip coating	N/A	2.69	N/A	SBF	N/A	^56^
PLA	Dip coating	−1.28	1.798	19.837	SBF	N/A	^57^
PCL/ZnO 3wt%	Electrospinning	−1.15	2.11	N/A	SBF	ZnO	^18^
PCL	Dip coating	−1.5	33.5	N/A	DMEM	N/A	^58^
SBF/ZrO_2_ NPs	Anodization	−1.41	3.17	N/A	SBF	N/A	^8^
Mg-PDA@TiO2	Dip coating	−1.28	2.04	N/A	PBS	N/A	^59^
Mg-PDA	Dip coating	−1.54	13.85	N/A	PBS	N/A	^59^
PLA/ZnO	Dip coating	−0.61	0.471	5.199	SBF	ZnO	^60^

^*^N/A This mean that not measured or has no effect on antibacterial.

### Immersion test

**Figure 5:**
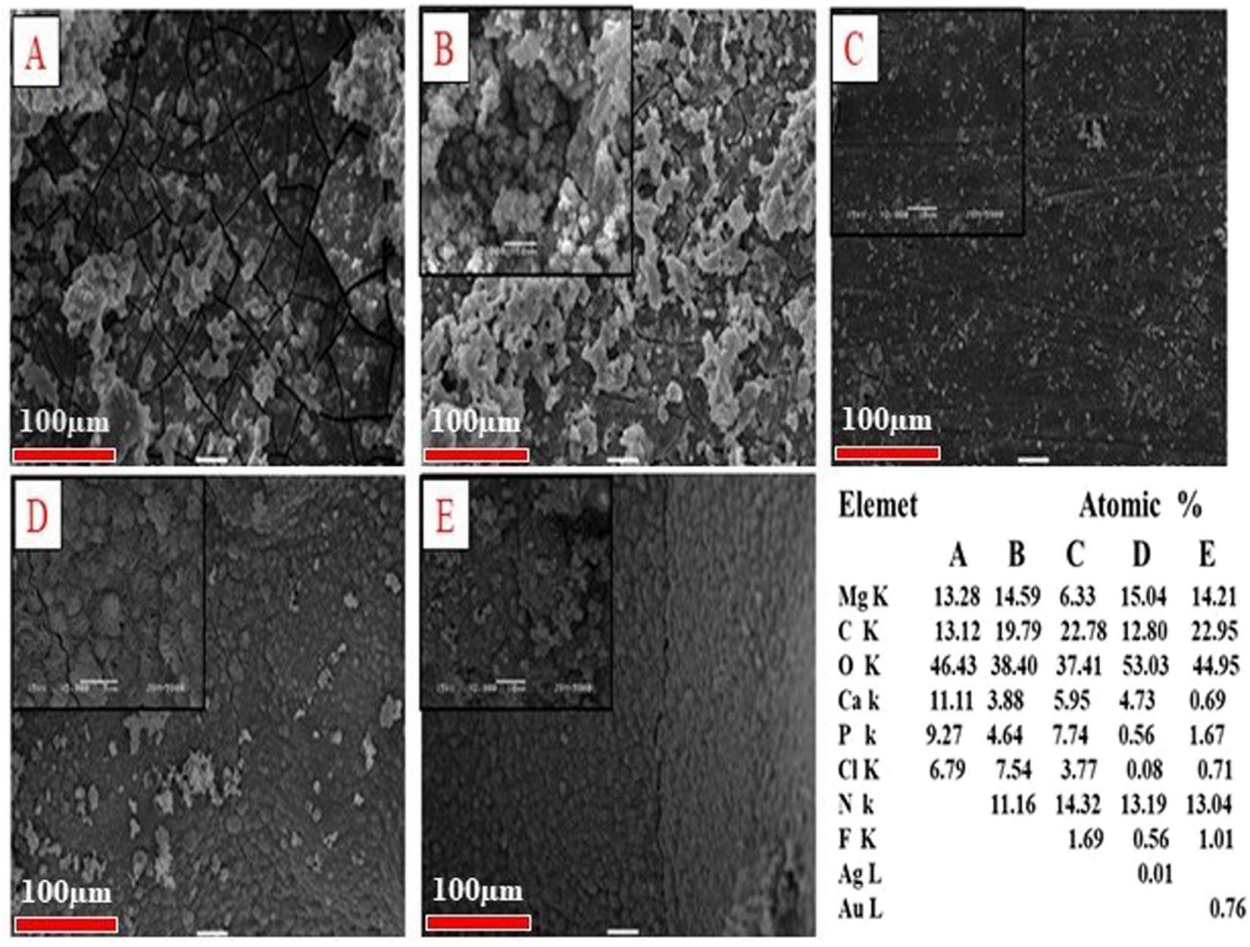
SEM images showing the morphology of a bare AZ31 alloy and different coatings after a 7-day immersion in an SBF solution at 37 °C and its elemental chemical composition via EDS. (**A**) Bare alloy, (**B**) PD coated sample, (**C**) HF- PD coated sample, (**D**) Ag HF-PD sample, and (**E**) Au HF-PD sample

The degradation behavior of the coated samples was also studied by immersing the samples in SBF solution. Figure 5 shows the SEM images of the different samples after immersing in SBF solution. The surface of the bare alloy show cracks due to the severe corrosion and the formation of the Mg(OH)_2_ layer^45^. Alternatively, the samples polymerized with PD on the alloy surface resulted in only a few cracks compared to bare alloy, and the HF pre-treated samples showed a more compact surface with small pores as a result of the coating of fluoride layer on the Mg alloy. The role of the corrosion product on the implant surface is also very important in terms of the interaction between the surface and the surrounding tissue. The EDX analysis that was performed at the surface resulted in the presence of the ions of Mg, O, C, N, P, F, Ca, and Cl. The presence of these ions indicate the formations of MgF_2_, magnesium chloride (MgCl_2_), Mg(OH)_2_, Mg phosphate, and Ca; however, the amounts and percentages of these phases depend on the surface pre-treatment and coating. The inset table in Fig. 5 is a summary of the composition that was obtained from the EDX analysis. The bare alloy shows the highest percentage of Cl, which was decreased in the case of the samples pre-treated with HF and coated with PD, and this is due to the Cl^−^ ion interaction with the Mg surface that forms MgCl_2_ as a corrosion product. Besides the presence of Ca, the presence of P is also indicated that will cause the formation of an apatite layer on the bare alloy surface which facilitate interaction with surrounding media and led to accelerate degradation process. In contrast, the formation of the composite coating act as barrier layer with ability to retard interaction and retard degradation process which confirmed by lower values from these elements, which can be explained by the presence of a successful protection film.

Immersion factors such as immersion solution pH value variation can indicate the corrosion behavior of the coated/uncoated samples. Biodegradability of Mg implant could be represented in chemical equation (3) in terms of pH values.

3 $${\rm{Mg}}+{{\rm{2H}}}_{{\rm{2}}}{\rm{O}}\to {{\rm{Mg}}}^{+2}+{{\rm{2OH}}}^{-}+{{\rm{H}}}_{{\rm{2}}}\,\uparrow $$

**Figure 6:**
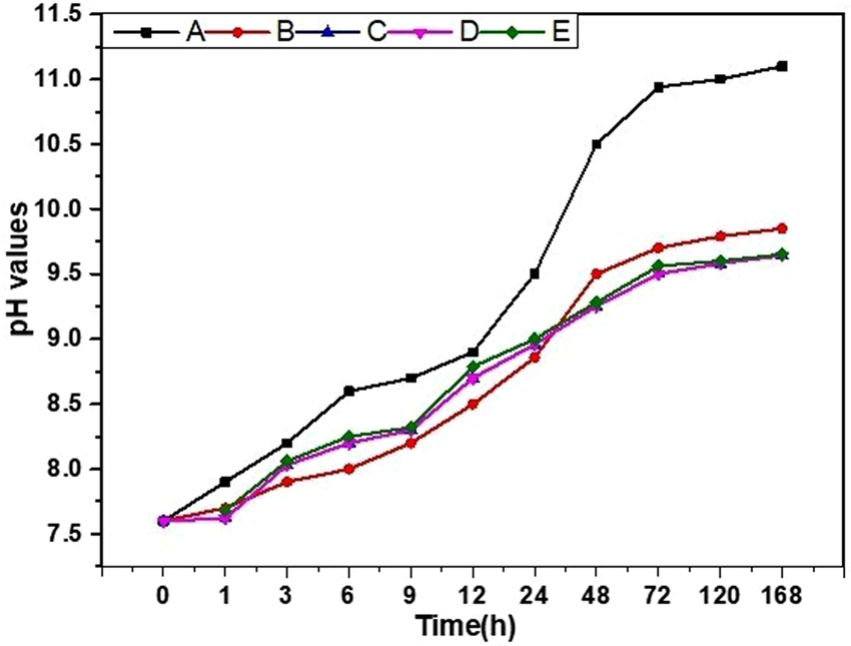
pH measurements of an SBF solution containing a bare AZ31 alloy and after different coatings at different time intervals. (**A**) Bare alloy, (**B**) PD coated sample, (**C**) HF- PD coated sample, (**D**) Ag HF-PD sample, and (**E**) Au HF-PD sample.

**Figure 7:**
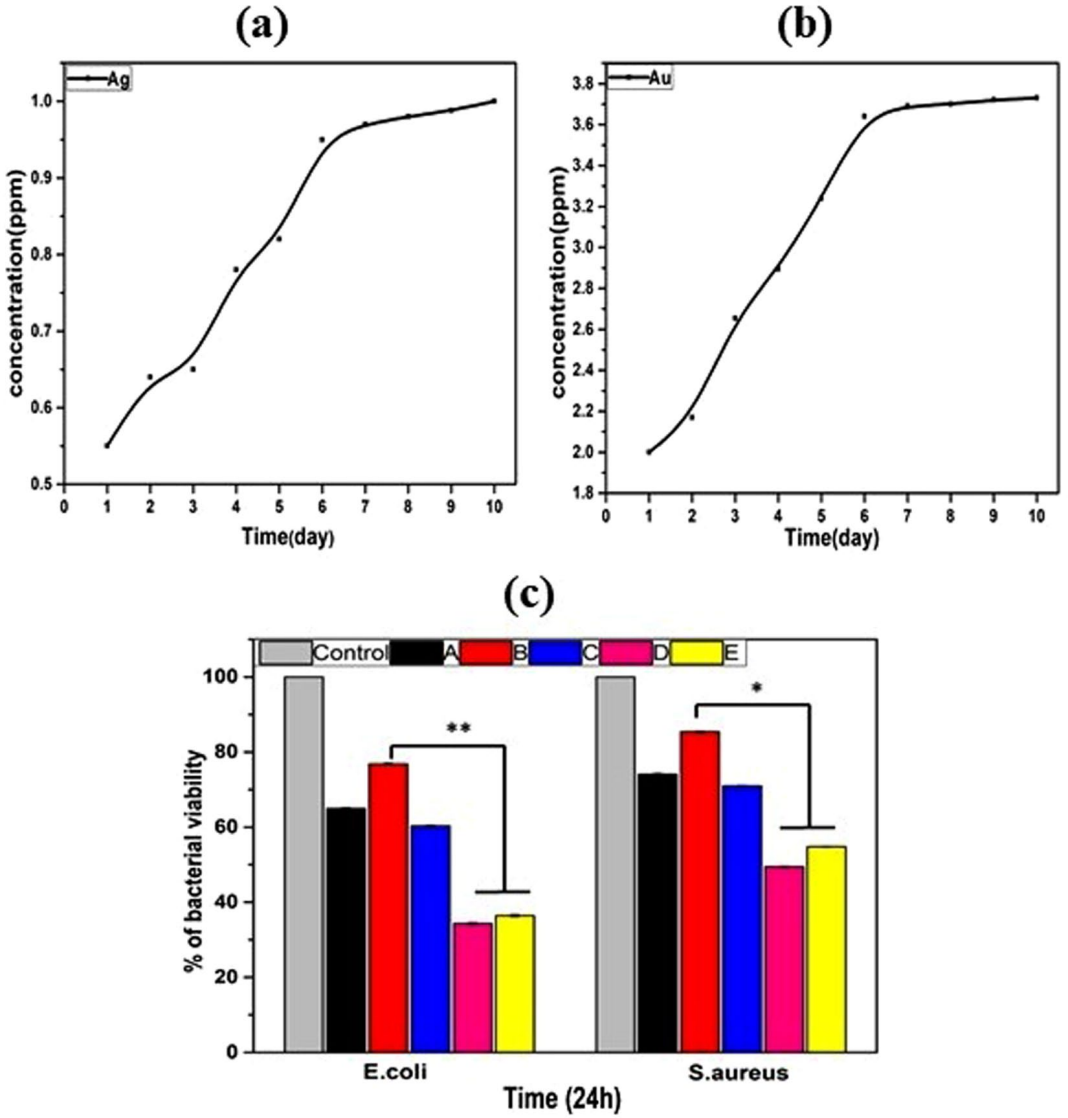
(**a**) shows Ag-ion release, (**b**) shows Au-ion release, Bacterial test: (**c**) shows bacteria-cell viability. (A) Bare alloy, (B) PD coated sample, (C) HF- PD coated sample, (D) Ag HF-PD sample, and (E) Au HF-PD sample. The data is reported as the mean ± SD (n = 3 and p < 0.05).

Hydroxide group represents the alkalinity of the sample when immersed in the SBF solution. Figure 6. shows the pH measurements in the SBF solution for different samples, where the pH reached the maximum value of 11 for the bare sample. After 7 days of immersion, this alkaline media showed a high degradation of the untreated sample, whereas the coated samples show a much better protection due to the coating effect that can delay the corrosion rate and result in a control of the pH^46^. Furthermore, the released Ag and Au ions were measured in the PBS solutions of the different samples at different time intervals, as shown in Fig. 7(a,b). The behaviors of the released ions show the increasing of the ions ppm values through the use of the ICP measurements until the steady-state values occurred after 10 days. The initial release occurred gradually, which is not toxic to the cells, as it provides a balance of the biocompatible and antibacterial activities.

### Antibacterial test

Clinical infections associated with surgical implants are generally more challenging to control owing to longer period of antibiotic therapies and frequent surgical procedures. Therefore, an effective combination of decent antibacterial function and acceptable cytotoxicity is what the clinic needs, which will be a balanced selection for biodegradable Mg based metals. Hence, the antibacterial effects of the different samples that were obtained from the spectrophotometric-data analysis (Fig. 7c). In this study, the bacterial effects of the bare alloy as a control and PD were compared, along with the HF-PD-coated samples with and without the Ag NPs and Au NPs. The resulting data showed the antibacterial effect of the bare sample against E. coli and S. aureus bacterial types, which explains the hindrance of the bacterial growth that is due to the generation of an alkaline medium in both solutions; this generation is from the degradation of the alloy that is facilitated by the surface corrosion. On the other hand, the PD-coated sample displayed a decreased antibacterial effect that confirmed an improved post coating corrosion resistance. The PD-coated sample after the HF treatment showed a bacterial inhibition due to the F ions, which exert an antibacterial effect^47^. The coating with the Ag and Au NPs showed the highest antibacterial effects, and this can be explained by the ability of the metal NPs to change the metabolite pathway and release mechanism of the bacterial cells, so that the ions can undergo an ion exchange between the imidazole and thiol groups of the bacteria, leading to a DNA malfunction that deactivates the replication ability of the bacteria^48,49^. Further, the bactericidal effect in the case of the E. coli is greater than that of the S. aureus, and this can be explained by the thicker cell wall of the S. aureus compared with the E. coli^50^. The results show a balance between the biocompatibility and antibacterial properties *in vitro*. The results also confirm that the presence of Ag and Au on the alloy-coating resulted in enhanced antibacterial effects against both the gram-positive and the gram-negative bacteria.

### Biocompatibility

**Figure 8:**
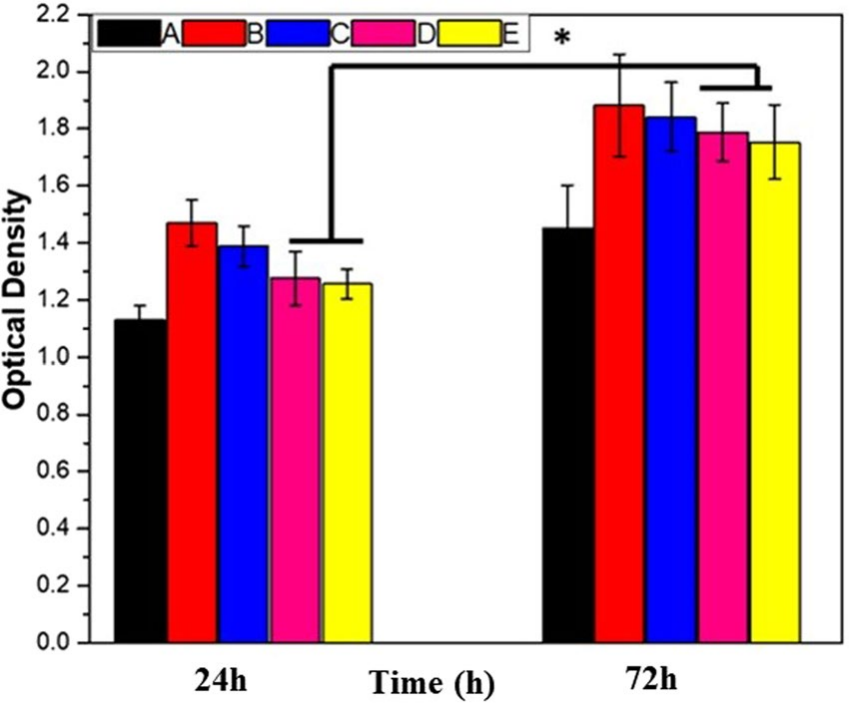
Cell viability of MC3T3-E1 cells in different extracts after 24-hr and 72-hr incubations. (A) Bare alloy, (B) PD coated sample, (C) HF- PD coated sample, (D) Ag HF-PD sample, and (E) Au HF-PD sample. The data is reported as the mean ± SD (n = 3 and p < 0.05).

**Figure 9:**
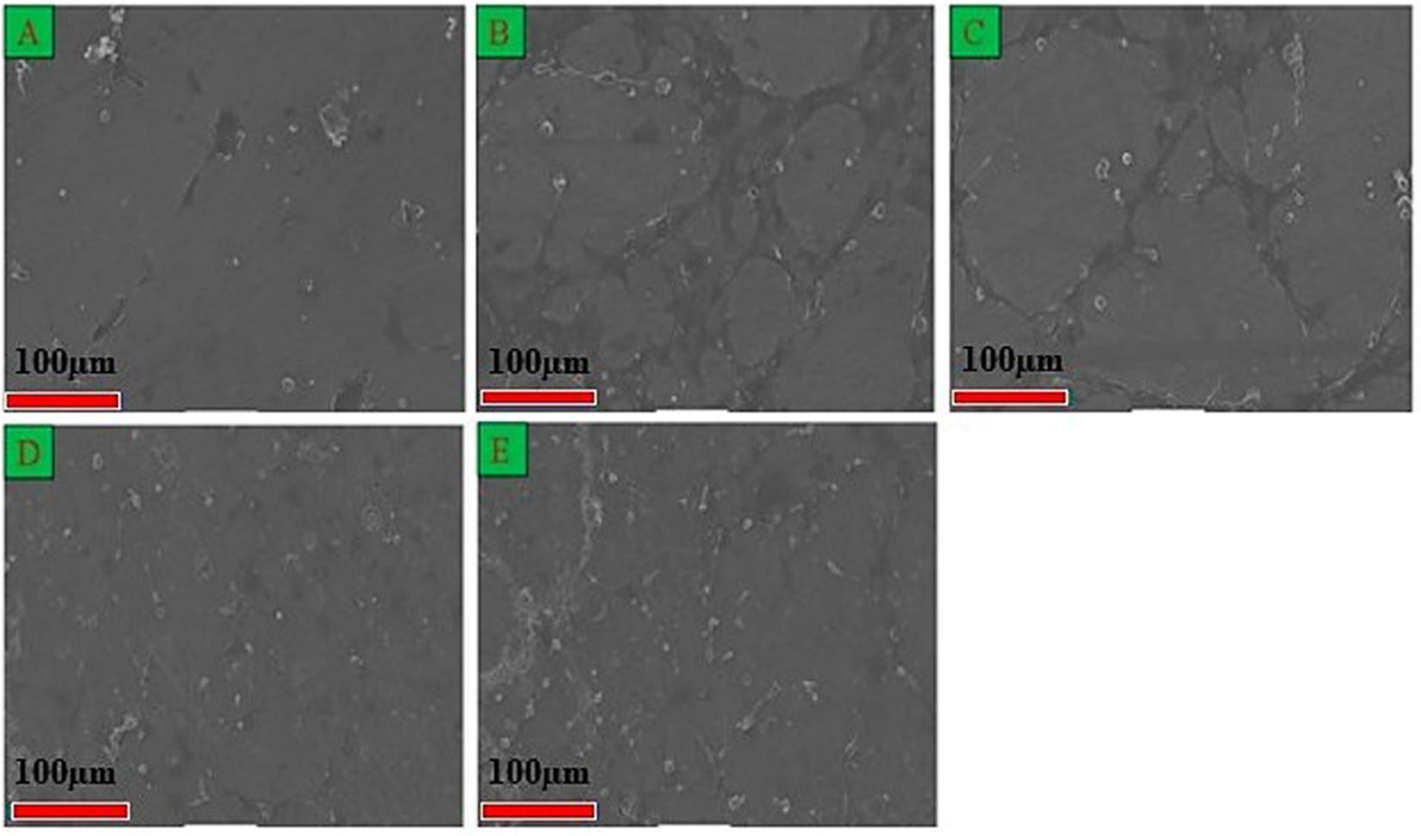
SEM morphologies of MC3T3-E1 cells on different surfaces after a 24-hr incubation. (**A**) Bare alloy, (**B**) PD coated sample, (**C**) HF- PD coated sample, (**D**) Ag HF-PD sample, and (**E**) Au HF-PD sample.

Biocompatibility plays a major role in the development of biomaterials. The *in vitro* cell viability is essential for the evaluation of the biocompatibility of the materials of the present study, and it was evaluated using the CCK-8 assay, as shown in Fig. 8. The biological response and biocompatibility of the bare and coated Mg-alloy samples were assessed using the cellular attachment and growth of MC3T3-E1 mouse osteoblast cells over 1 day. It has been found that the samples coated with PD displayed the highest cell proliferation, thereby revealing the biocompatibility of PD. The mechanism behind the cell proliferation on PD layer could be contributed to the quinone group on PDA coatings induced a larger amount of protein adsorption, and subsequently promoted cell attachment and proliferation^38^. The HF-PD coated and the Ag and Au NPs loaded samples still showed higher values than the bare alloy, strongly indicating that the coating layer promote the osteoblastic cellular proliferation. MgF_2_ is an insoluble compound on the alloy surface which improve the corrosion resistance without any cytotoxicity as reported earlier for showing the osteoblastic cell response on fluoridated hydroxyapatite coatings^9,38,51,52^. Further confirmation of the cell proliferation and adhesion has been carried out by performing the SEM of the cell-seeded surfaces of different samples. Figure 9 shows SEM image showing the interaction between the cell and the material substrates of different samples. On the surface of the bare alloy, only a few cells were observed as attached cells, confirming that the alloy surface cannot provide a suitable substrate for the cells to adhere due to the cracks that are present on its surface. Alternatively, after the polymerization of dopamine on the alloy surface, the bare alloy displayed an enhanced cellular adhesion and growth, as shown in Fig. 9 Consequently, after the treatment of the surface with HF and the PD coating and the Ag and Au NPs formation still showed a considerable cellular attachment and proliferation. The SEM images and cell-viability assay results explain that the coating layer on the Mg samples improves the biocompatibility; furthermore, the incorporation of the metal NPs on the PD coating did not considerably affect the cellular behavior on the Mg substrates, indicating that the coatings containing proper concentrations of the Ag NPs and Au NPs could be acceptable in biomedical applications. The results indicate that the samples loaded with the Ag and Au NPs provided slow and moderate ion releases, thereby achieving a balance between the antibacterial and biocompatible properties.

## Conclusion

PD was successfully polymerized on a HF-pre-treated AZ31-alloy surface using a simple and easy immersion method. The uniform layer of PD on the alloy surface resulted in the *in-situ* reduction of Ag and Au NPs on the surface. The composite coating improved the corrosion resistance of the bare alloy in an SBF solution with a remarkable decrease of the corrosion-current density compared with the bare alloy, moreover has a broad antibacterial effect against the E. coli and S. aureus species, thereby achieving a balance with the biocompatibility that is shown in the cell-culture test. This kind of multifunction composite coating needs greater attention in terms of the surface modification of bio-resorbable Mg and its alloys in degradable implant application.

## Experimental

### Materials and sample preparation

The samples of the AZ31 Mg alloy (Alfa Aesar Company, Republic of Korea) were cut into the dimensions of 12 × 12 × 6.35 mm to fit them into the holder of the electrochemical cell for the corrosion evaluation. The chemical composition was balanced as Al 3%, Zn 1%, and Mg 96%. Further, these samples were polished with 200, 800, 1200, and 2000 silicon carbide (SiC) abrasive papers, followed by their immersion in ethanol, a 15-min sonication, a washing with distilled water, and then a drying under hot air. Silver nitrate (AgNO_3_), gold chloride trihydrate (HAuCl_4_.3H_2_O), dopamine hydrochloride, and Hank’s balanced salts were purchased from Sigma Aldrich (USA); tris (hydroxy methyl) amino methane (TRIS) 99.0% and the HF were purchased from Samchun (Republic of Korea); sodium chloride (NaCl) was purchased from DaeJung Co., Ltd. (Republic of Korea); and potassium chloride (KCl) was purchased from Showa Co., Ltd. (Japan).

### Coating procedure

The AZ31 alloy was coated with the PD using a simple dip-coating method. Briefly, a (50 mL) solution of NaCl (0.4 g), KCl (0.01 g), TRIS (0.15 g), and dopamine hydrochloride (0.02 g/mL) was prepared under a nitrogen (N) environment. The coating has been carried out by immersing the AZ31 alloy in 20 mL of the immersion solution and keeping in the shaker for 3 h at 37 °C and by this time the surface color change was also noted. A different coating condition was achieved by pre-passivating the samples in 48 wt.% HF for 24 h before the coating of the PD layer as described above. The HF was used for the formation of a protective layer of magnesium fluoride (MgF_2_), which delays the occurrence of corrosion due to the presence of the Cl ion in the immersion solution. In order to reduce the Ag and Au Nps, the HF-PD coated samples were separately kept in 40 mL of distilled water containing the previous weighted Ag nitrate and Au chloride trihydrate particles for 2 h in a shaking incubator. Later, the samples were washed with distilled water to remove the unattached particles, and then they were dried under in a heater.

### Coating Characterization

The surface morphology of the samples was investigated using a SEM in the field-emission mode for which a high voltage of 15 kV was applied. The chemical compositions of the samples were evaluated using the XPS and energy dispersive X-ray (EDS) analysis. Aluminium (Al) was used as the anode material of the Thermo/K-Alpha ESCA System. Moreover, the elemental mapping and the composition of the surface were investigated using an energy dispersive spectrometer. Phase analyses of the coated film were measured using XRD (Rigaku, Japan). The average size of the nanoparticles was analyzed using Image J (NIH, USA) software.

### *In vitro* degradation study

An electrochemical corrosion test was performed using a ZIVE SP1 potentiostat/galvanostat device that had been connected to a three-electrode cell containing a simulated body fluid (SBF) solution, which is similar to physiological fluid. Linear-sweep potentiodynamic measurements were recorded with Ag/silver chloride (AgCl) saturated in KCl, platinum (Pt), and a sample serving as the reference, counter, and working electrodes, respectively. The current and voltage ranges are 10 µA and 10 V, respectively, and the initial (E_0_) and final (E_1_) potentials during the sweep measurements are −2500 mV and 1500 mV, respectively, with a scan rate of 5.0 mV/s. The surface area of the sample exposed to the solution is 0.875 cm^2^. All the parameters that were derived using the ZIVE SM Smart Manager Software Ver. 5.503 and the IVMAN Ver. 1.2 testing software are listed in Table 1.

The standard immersion test was performed by immersing the as prepared samples in the SBF solution^42^, Briefly commercial Hank’s balanced solution (Aldrich, H2387-1, without calcium, sodium and magnesium) mixed with MgSO_4_ (0.097 g), NaHCO_3_ (0.35 g), and CaCl_2_ (0.185 g) in 1 L distilled water and the pH of the solution was maintained at 7.4. The ratio of the surface area to the solution volume was kept as 1:30 cm^2^/mL^53^. The immersion test was performed thereafter for 7 days at 37 °C, the surface morphology was investigated using the SEM, and the chemical elemental compositions were evaluated using EDS. Further, the sample biodegradability was analyzed in terms of the pH values of the extraction that were determined at different time intervals. Furthermore, an inductively coupled plasma-optical emission spectroscopy (ICP-OES) instrument was used to measure the amounts of the Ag and Au ions that were released from the bare and coated samples; all of the samples were immersed in a phosphate buffered saline (PBS) solution, which was further diluted before the measurements were taken at different immersion-time intervals, for the inductively coupled plasma (ICP) test.

### Antibacterial test

The antibacterial activities of the samples were tested using both gram-positive (Staphylococcus aureus ATCC 29231) and gram-negative (Escherichia coli ATCC 52922) bacteria, which were used as model organisms. The antibacterial properties of the samples against each strain were determined using the spectrophotometric method. Briefly, bacterial cells were grown in Luria-Bertani broth (LB broth, pH 7.2) and incubated at 37 °C for 24 h. Bacterial inoculums were diluted in 20 mL of the LB-broth solution to obtain 10^7^ CFU/mL bacterial cells. The sterile samples were immersed in the bacterial suspension and incubated for 24 h. An LB broth containing the S. aureus and E. coli (10^7^ CFU/mL) was set as the control. Each 100 μL solution from all of the conditions was transferred into a 96-well plate, and the absorbance was measured using an iMarkTM Microplate reader at the wavelength of 620 nm. The cell viability of the bacterial cell in each sample is expressed as a percentage of that of the control.

### Biocompatibility test

An *in vitro* cell-culture test was performed using Dojindo’s cell-counting kit-8 (CCK-8) assay to study the cell proliferation on the different-coating samples. The sample extracts were prepared by immersing the different substrates in each 20 mL complete-culture medium (α-MEM, supplementary with 10% fetal bovine serum and 1% penicillin streptomycin), followed by an incubation at 37 °C for 24 h. Preosteoblast cells (MC3T3-E1, 1 × 10^4^ cells/100 μL) were seeded in a 96-well plate with the culture medium and incubated at 37 °C under 5% carbon dioxide (CO_2_) for 24 h. The culture medium was later replaced by the extracts and further incubated for 1 and 3 days. Then, 10 μL of the CCK-8 solution was added to each well and they were incubated for 4 h. Later, the absorbance was measured using the iMarkTM Microplate reader at the wavelength of 450 nm. In addition, the morphological behavior of the cells on the surface of the samples was observed using the SEM. The samples were sterilized under a UV ray for 24 h, followed by a rinsing in ethanol. The samples were then placed in 12-well plates and the preosteoblast cells (MC3T3-E1, 1 × 10^4^ cells/well) were seeded directly and incubated at 37 °C under 5% CO_2_ for different times. Later, the samples were washed with the PBS and fixed in 3.5% glutaraldehyde for 1.5 h. The dehydration of the sample was performed using a series of ethanol solutions (25%, 50%, 75%, and 100%). Lastly, the dried samples were observed using the SEM.

## Electronic supplementary material

supplementary information
