# Supplementary material for: Strategic design of a Mussel-inspired in situ reduced Ag/Au-Nanoparticle Coated Magnesium Alloy for enhanced viability, antibacterial property and decelerated corrosion rates for degradable implant Applications
Source: Sci Rep. 2019 Jan 15;9:117. doi: 10.1038/s41598-018-36545-3 (PMC6333833; doi:10.1038/s41598-018-36545-3)
Supplement: Supplementary file 1 — supplementary information [file 41598_2018_36545_MOESM1_ESM.docx]

**Strategic design of a Mussel-inspired in situ reduced Ag/Au-Nanoparticle Coated Magnesium Alloy for enhanced viability, antibacterial property and decelerated corrosion rates for degradable implant Applications**

Abdelrahman I. Rezk^1^, Arathyram Ramachandra Kurup Sasikala ^1, 3*^, Amin Ghavami Nejad^1^, Hamouda M. Mousa^1, 2^, Young Min Oh^4^, Chan Hee Park^1, 3*^ and Cheol Sang Kim^1, 3*^

^1^ Department of Bionanosystem Engineering, Chonbuk National University, Jeonju, Jeonbuk 561-756, Republic of Korea,

^2^ Department of Engineering Materials and Mechanical Design, Faculty of Engineering, South Valley University, Qena 83523, Egypt.

^3^ Division of Mechanical Design Engineering, Chonbuk National University, Jeonju, Jeonbuk 561-756, Republic of Korea.

^4^ Department of Neurosurgery, Chonbuk National University Medical School & Hospital, Chonbuk National University, Jeonju, Jeonbuk 561-756, Republic of Korea.

*Corresponding authors:

Tel: +82 63 270 4284, Fax: +82 63 270 2460

[chskim@jbnu.ac.kr](mailto:chskim@jbnu.ac.kr) (Cheol Sang Kim)

[biochan@jbnu.ac.kr](mailto:biochan@jbnu.ac.kr) (Chan Hee Park)

[arathy@jbnu.ac.kr](mailto:arathy@jbnu.ac.kr) (Arathyram Ramachandra Kurup Sasikala)


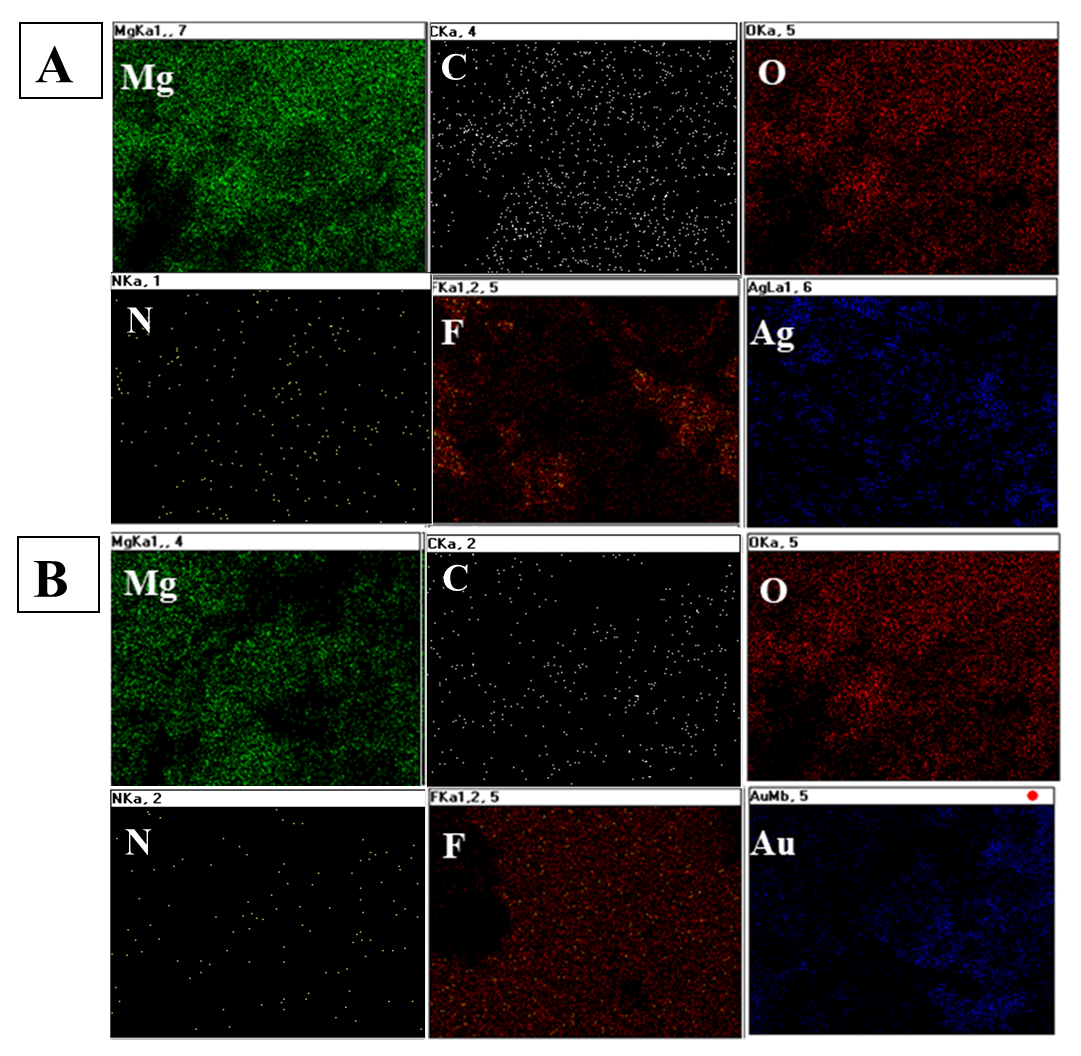


**Figure S1:** EDS elemental mapping showing the composition in A) Ag HF-PD sample, and E) Au HF-PD sample**.** Green is corresponding to (Mg), grey is corresponding to (C), red is corresponding to (O), yellow is corresponding to (N), orange is corresponding to (F), blue is corresponding to (Ag) in Fig. A and in Fig. B Blue is corresponding to (Au) respectively).

**Figure S2:** FESEM images display nanoparticle on the surface of the alloy A is Ag HF-PD sample, and (B) is Au HF-PD.


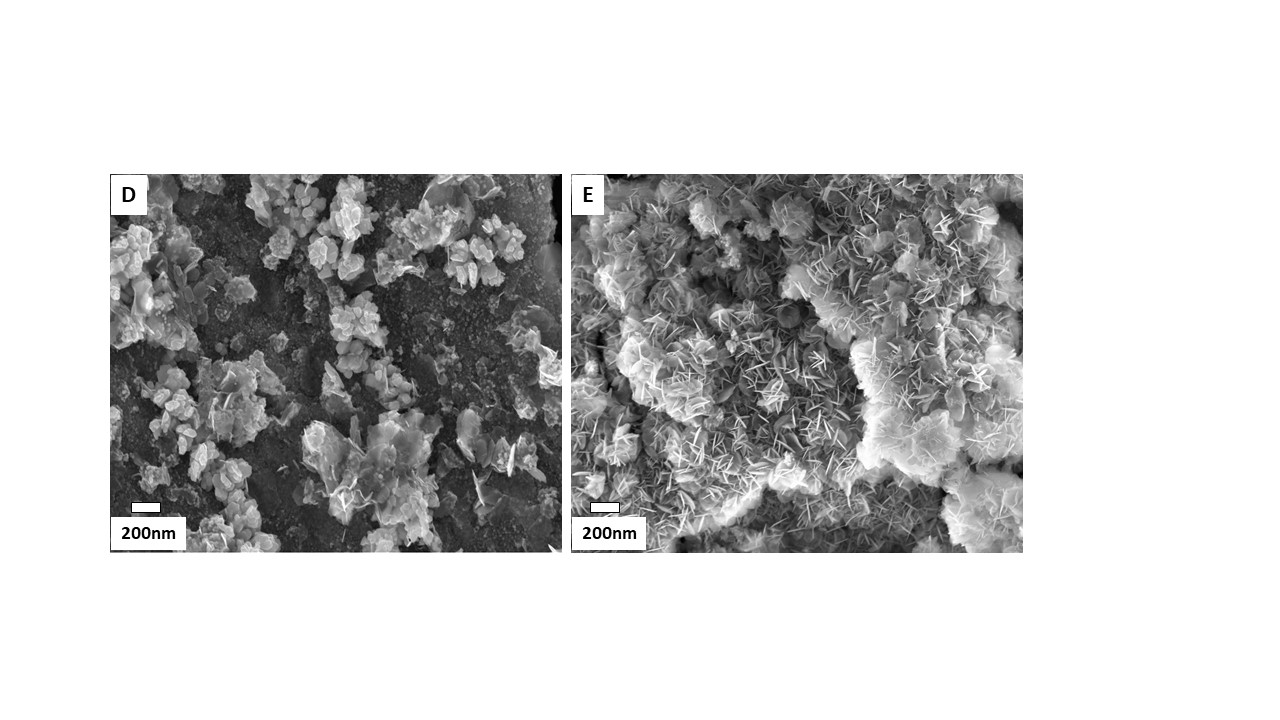


**A**

**B**


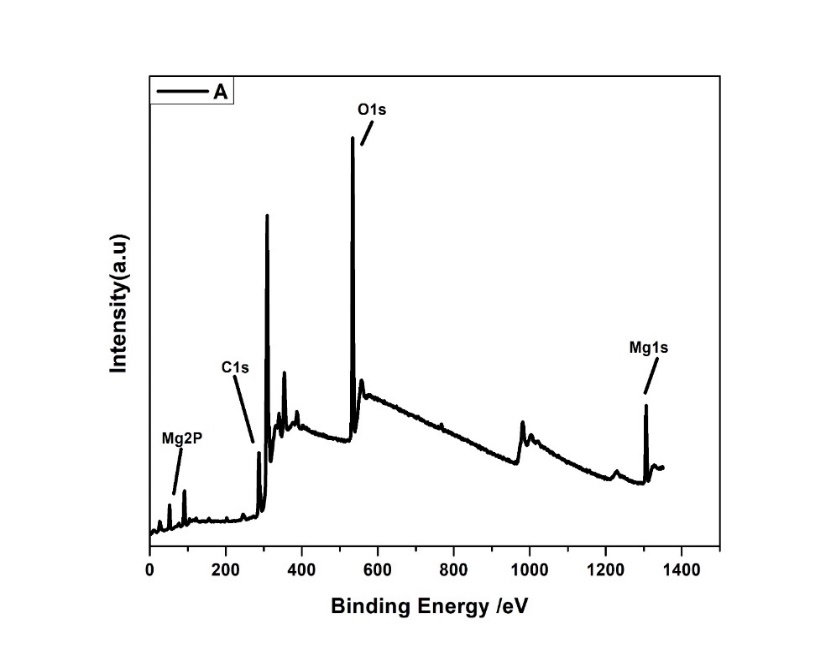

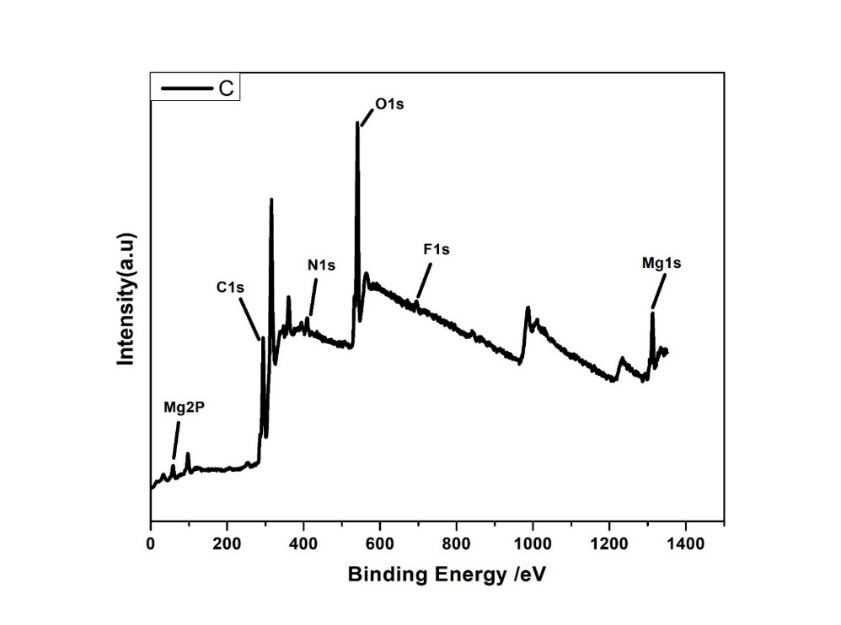

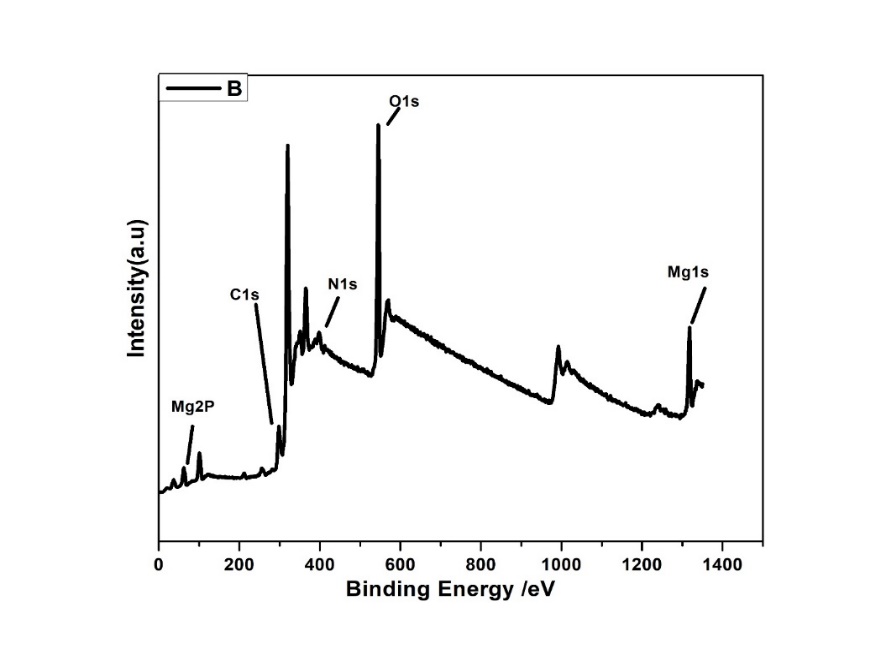

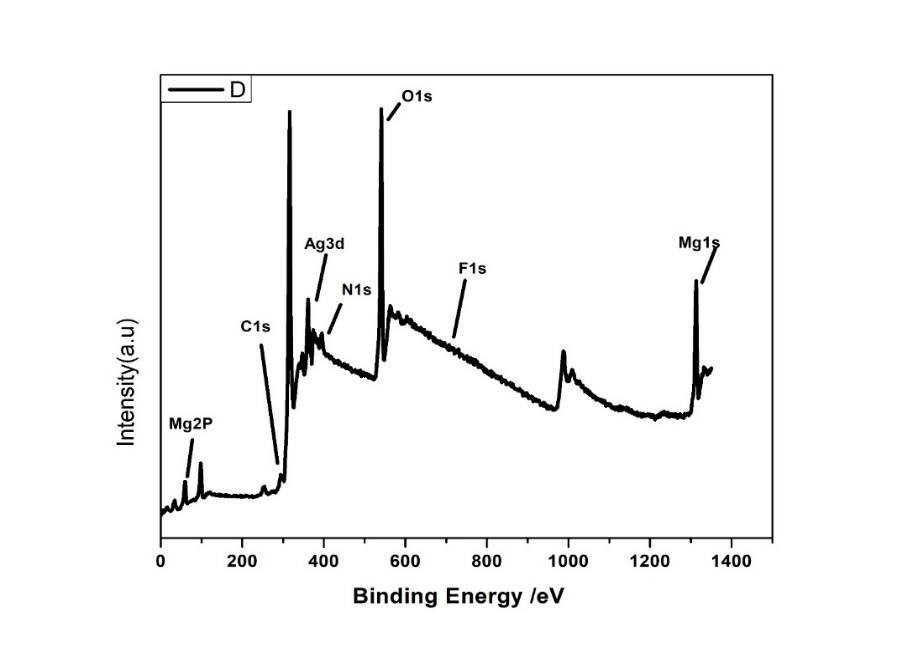

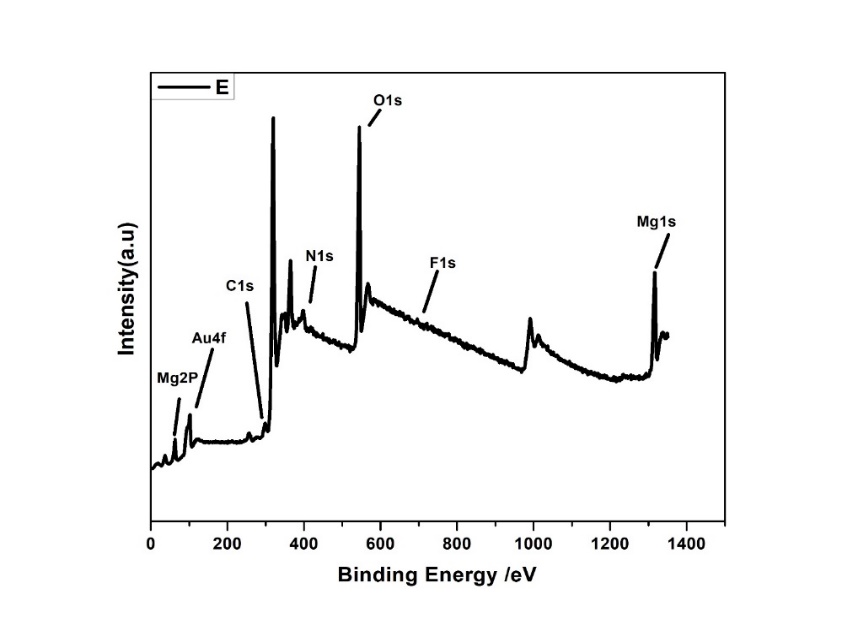

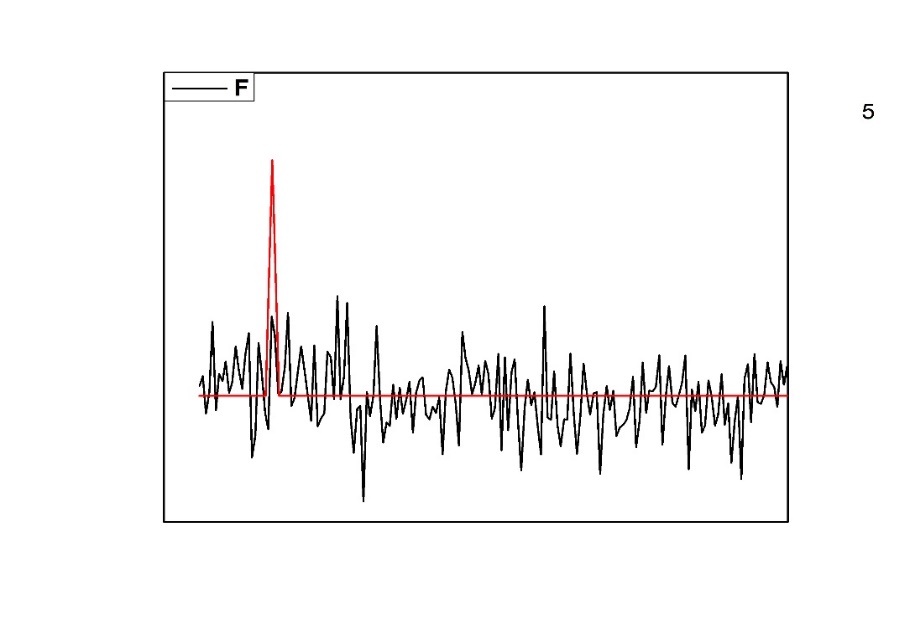


Figure S3. XPS full spectra and narrow-scan spectra of Ag and Au elements. (A) Bare alloy, (B) PD coated sample, (C) HF- PD coated sample, (D) Ag HF-PD sample, and (E) Au HF-PD sample, (F) peak of F element.


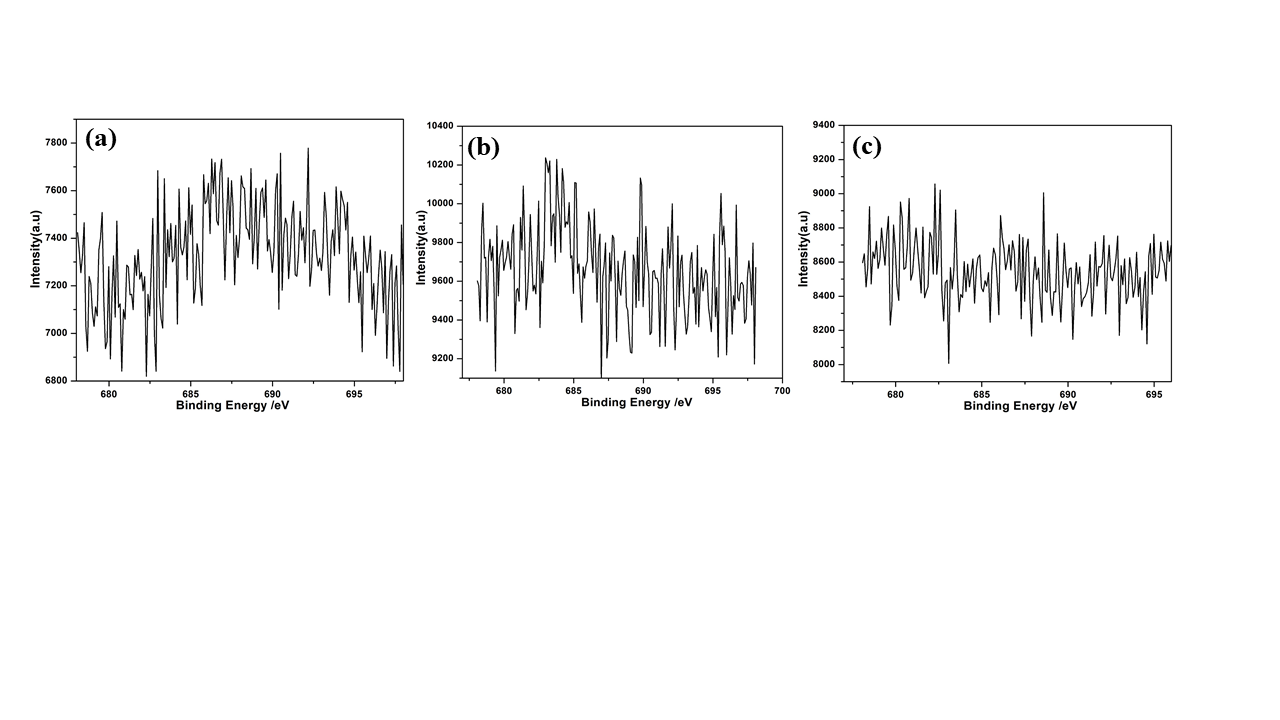
**Figure S4:** XPS full spectra and narrow-scan spectra of Ag and Au elements. (a) HF- PD coated sample, (b) Ag HF-PD sample and (c) Au HF-PD sample.


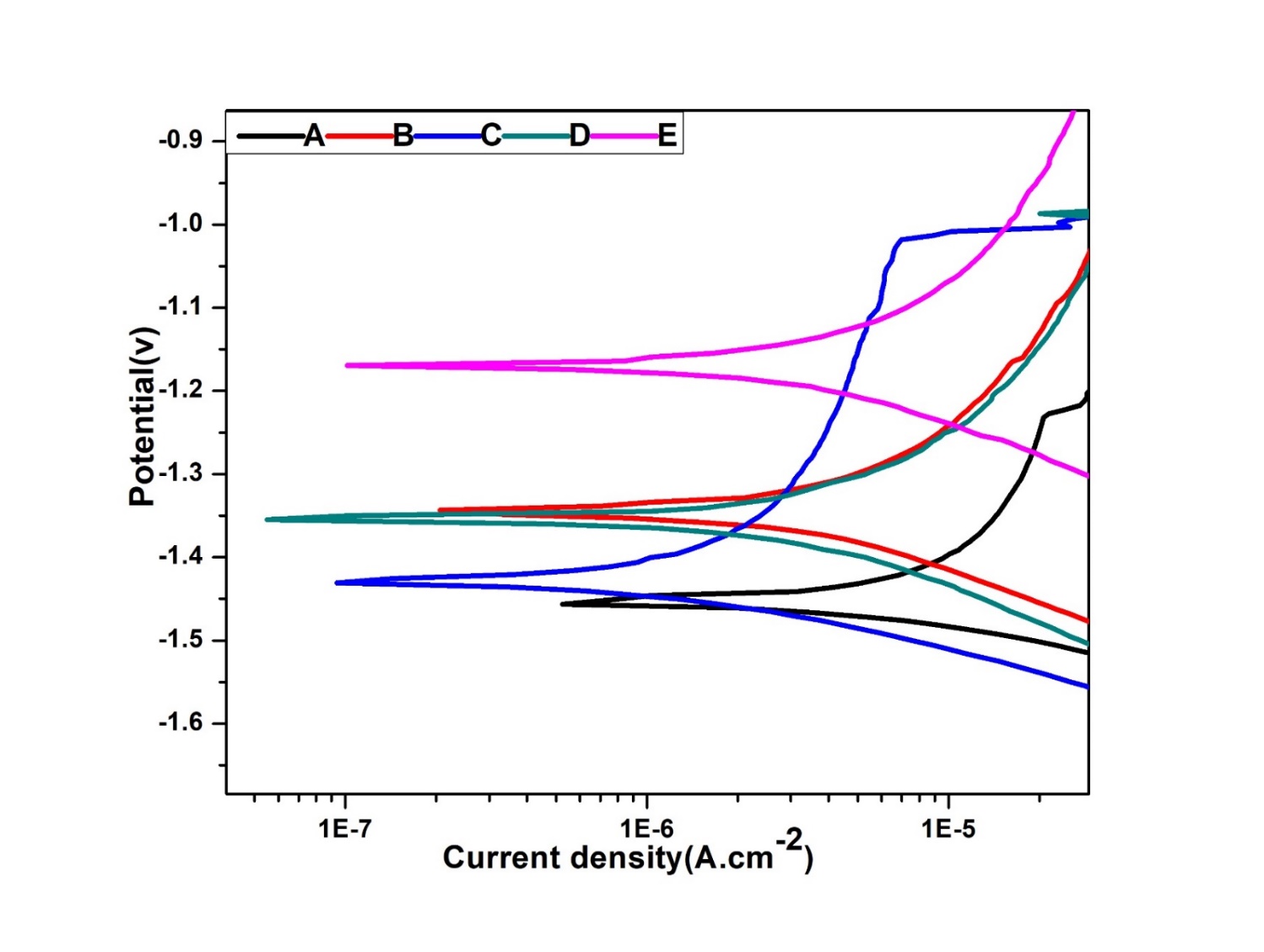


**Figure S5.** Magnified Potentiodynamic-polarization curves of a bare alloy and different coated samples. A) Bare alloy, B) PD coated sample, C) HF- PD coated sample, D) Ag HF-PD sample, and E) Au HF-PD sample.
